# A computerized facial approximation method for *Homo sapiens* based on facial soft tissue thickness depths and geometric morphometrics

**DOI:** 10.1111/joa.13920

**Published:** 2023-06-27

**Authors:** Wuyang Shui, Xiujie Wu, Mingquan Zhou

**Affiliations:** ^1^ Department of Archaeology University of York York UK; ^2^ School of Information Science and Technology Northwest University Xi'an China; ^3^ Key Laboratory of Vertebrate Evolution and Human Origins of Chinese Academy of Sciences Institute of Vertebrate Paleontology and Paleoanthropology Chinese Academy of Sciences Beijing China; ^4^ CAS Center for Excellence in Life and Paleoenvironment Beijing China

**Keywords:** covariation, craniofacial relationships, facial approximation, geometric morphometrics, soft tissue thickness depths

## Abstract

Facial approximation (FA) provides a promising means of generating the possible facial appearance of a deceased person. It facilitates exploration of the evolutionary forces driving anatomical changes in ancestral humans and can capture public attention. Despite the recent progress made toward improving the performance of FA methods, a limited understanding of detailed quantitative craniofacial relationships between facial bone and soft tissue morphology may hinder their accuracy, and hence subjective experience and artistic interpretation are required. In this study, we explored craniofacial relationships among human populations based upon average facial soft tissue thickness depths (FSTDs) and covariations between hard and soft tissues of the nose and mouth using geometric morphometrics. Furthermore, we proposed a computerized method to assign the learned craniofacial relationships to generate a probable facial appearance of *Homo sapiens*, reducing human intervention. A smaller resemblance comparison (an average Procrustes distance was 0.0258 and an average Euclidean distance was 1.79 mm) between approximated and actual faces and a greater recognition rate (91.67%) tested by a face pool indicated that average dense FSTDs contributed to raising the accuracy of approximated faces. Results of partial least squares (PLS) analysis showed that nasal and oral hard tissues have an effect on their soft tissues separately. However, relatively weaker RV correlations (<0.4) and greater approximation errors suggested that we need to be cautious about the accuracy of the approximated nose and mouth soft tissue shapes from bony structures. Overall, the proposed method can facilitate investigations of craniofacial relationships and potentially improve the reliability of the approximated faces for use in numerous applications in forensic science, archaeology, and anthropology.

## Graphical abstract

We quantify detailed craniofacial relationships between facial bone and soft tissues and present a computerized facial approximation method. A smaller resemblance comparison and a greater recognition rate demonstrate that average facial soft tissue thickness depths at landmarks and semilandmarks contribute to raising the accuracy of approximated faces. Relatively weaker correlations and greater approximation errors suggest that the approximated nose and mouth soft tissue shapes need to be used with caution.

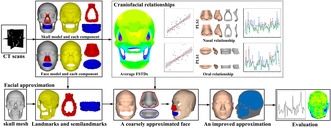

## 1 INTRODUCTION

Facial approximation (FA), also known as facial reconstruction, is a commonly used technique to recreate the probable facial appearance of a deceased person from skeletal remains. In the absence of other evidence, it has served as a method of last resort to recover possible facial likenesses for use in triggering memories to aid forensic identification of a seriously decomposed cadaver (Baldasso et al., 2021; Nelson & Michael, 1998). Additionally, it seeks to recreate sculptural portraits of undocumented *Homo sapiens*, for example, famous historical figures and our recent ancestors, in the realms of archaeology and anthropology (Benazzi et al., 2009; Marić et al., 2020). The approximated faces provide new insights into understanding the characteristic features of human fossils, exploring the evolutionary forces driving anatomical changes in ancestral humans and capturing public attention. Until recently, FA methods for creating three‐dimensional (3D) facial appearances have involved manual, virtual sculpture, and computer‐based techniques.

The conventional 3D manual FA method is primarily based upon clay sculpturing techniques over skull casts, which have been used to approximate hundreds of facial sculptures of *Homo sapiens* (Hayes, 2016). This method can be divided into three main approaches (Verzé, 2009): the American method based on facial soft tissue thickness depths (FSTDs) at landmarks, the Russian method based on muscle knowledge, and the combined Manchester method that is the most commonly used in forensic and archaeology fields. However, it is argued the approximated results are dependent upon the anatomical interpretation of craniofacial relationships between bony structures and facial morphology, and the artistic skill of the individual practitioner (Campbell et al., 2021; Stephan, 2015). For example, subjective and artistic interpretation may lead to biased and inconsistent results, and hence the practitioner requires anthropological and artistic training and experience in practice. Additionally, the manual method always lacks the standard procedure and is a time‐consuming task (Claes et al., 2010; Guyomarc'h et al., 2014).

To address the shortcomings of the manual method, a 3D interactive computerized method was developed by graphics software (e.g., ZBrush and Blender) and haptic feedback devices, mimicking the conventional Manchester method in the computer environment (Wilkinson et al., 2006). It has been successfully used in different case studies in archaeology and forensic science (Hamre et al., 2017). In practice, several virtual pegs representing FSTDs are attached to the surface of the digital skull at landmarks, and then pre‐modeled facial muscles are interactively placed onto the skull. A critical point is to ensure altered muscles meet the specifications of the particular skull following anatomical guidelines. Subsequently, the appropriate facial features (e.g., eyes, nose, mouth, and ears) are chosen from a dataset to place over the facial model. Skin layers are added over the muscle structures and the detailed morphology and features are sculpted to improve the realism of the approximated faces. Such a method offers workflow standardization, and the pre‐modeled muscles and facial features provide valuable anatomical guidance to reduce approximation bias and subjective interpretation. Nevertheless, such a system still involves a degree of subjective interpretation and requires modeling skills training.

Progress in the development of medical imaging technologies, such as computed tomography (CT), cone‐beam computed tomography (CBCT), and magnetic resonance imaging (MRI), has led to the development of semi‐ and fully‐automatic computerized FA methods (Claes et al., 2010; Wilkinson, 2005). The earliest proposed 3D computerized FA efforts involved the deformation of the template face, for example, an average human face, to fit the estimates of facial points generated by assigning average FSTDs at landmarks with regard to ethnicity, sex to the dry skull (Vanezis et al., 2000). However, the limited number of facial points cannot adequately capture detailed morphology, and hence personal experience and subjective imagination are inevitable. Recent studies have employed FSTDs at landmarks and high‐density semilandmarks to build a facial envelope with better results (Gietzen et al., 2019; Shui et al., 2016). To avoid the very substantial computation costs of dense FSTDs, an alternative deformation‐based computerized FA method has been developed based on the deformation of the template face followed by transformation to warp the template skull to the dry skull (De Buhan & Nardoni, 2018; Quatrehomme et al., 1997; Turner et al., 2005). Nonetheless, the ultimate approximation result might resemble the template face and a large skull registration error adversely affects the accuracy of the approximation (Deng et al., 2011; Wilkinson, 2005).

Since a large collection of skull and face models facilitates the investigation of detailed craniofacial relationships amongst human populations to achieve anatomical modeling, several recent studies used machine learning algorithms, for example, multiple regression, to learn the relationships of principal component (PC) scores between 3D skulls and faces in a holistic way, and then applied them to the dry skull to generate the ultimate approximation (Berar et al., 2011; Madsen et al., 2018; Shui et al., 2017). Furthermore, deep learning methods, for example, generative adversarial nets, were developed to generate craniofacial images and 3D meshes from 2D skull images (Li et al., 2022; Zhang et al., 2022). Nevertheless, facial morphology is complex and different regions of the head exhibit different morphological changes in craniofacial relationships (Deng et al., 2016; Guyomarc'h et al., 2014), such that these holistic methods probably cannot provide detailed relationships in facial features. To tackle the issue, the skull and face are segmented into different components, and covariations between hard and soft tissues of each component are regressed to predict soft tissue shapes (Deng et al., 2016; Guyomarc'h et al., 2014).

Anatomical modeling to represent overall craniofacial relationships, and determination of the shape, size, and locations of facial features are the fundamental steps in enhancing the accuracy of FA (Guyomarc'h et al., 2014; Wilkinson, 2010). In this study, we present a computerized method for exploring detailed craniofacial relationships between bony structures and facial soft tissues and afterward developing a coarse‐to‐fine method to generate a probable facial appearance. A quantitative method based on resemblance comparison between approximated and actual faces and recognition rate tested by a face pool was used to validate the accuracy of the proposed method.

## 2 MATERIALS AND METHODS

### 2.1 Materials

In our previous studies (Deng et al., 2016; Shui et al., 2017), we constructed a skull and face dataset of modern living humans exhibiting normal morphological features without prior orthodontic treatment. Medical images of each individual were acquired using a clinical multi‐slice CT scanner system (Siemens Sensation 16) and then CT images were archived as standard DICOM 3.0 files of resolution 512 × 512. Each head within the database comprised digital models of the skull and face with personal information such as age, sex, and body mass index (BMI). To reduce the computational cost of morphological analysis, we extracted the external surfaces of skull and face of each individual. Each skull comprised more than 160,000 vertices and 320,000 triangle meshes, and each face consisted of more than 250,000 vertices and 500,000 triangle meshes. We manually placed skull landmarks left porion (*Lp*), right porion (*Rp*), left orbitale (*Lo*), and glabella (*G*), and transformed them into the common Frankfort coordinate system. The nonrigid registration method was used to establish dense point correspondences between skulls and faces separately. Thereafter, the average male skull and facial form (including shapes and sizes) surfaces were estimated by averaging dense point correspondences and used as templates to yield semilandmarks among skulls and faces in the present study.

**FIGURE 1:**
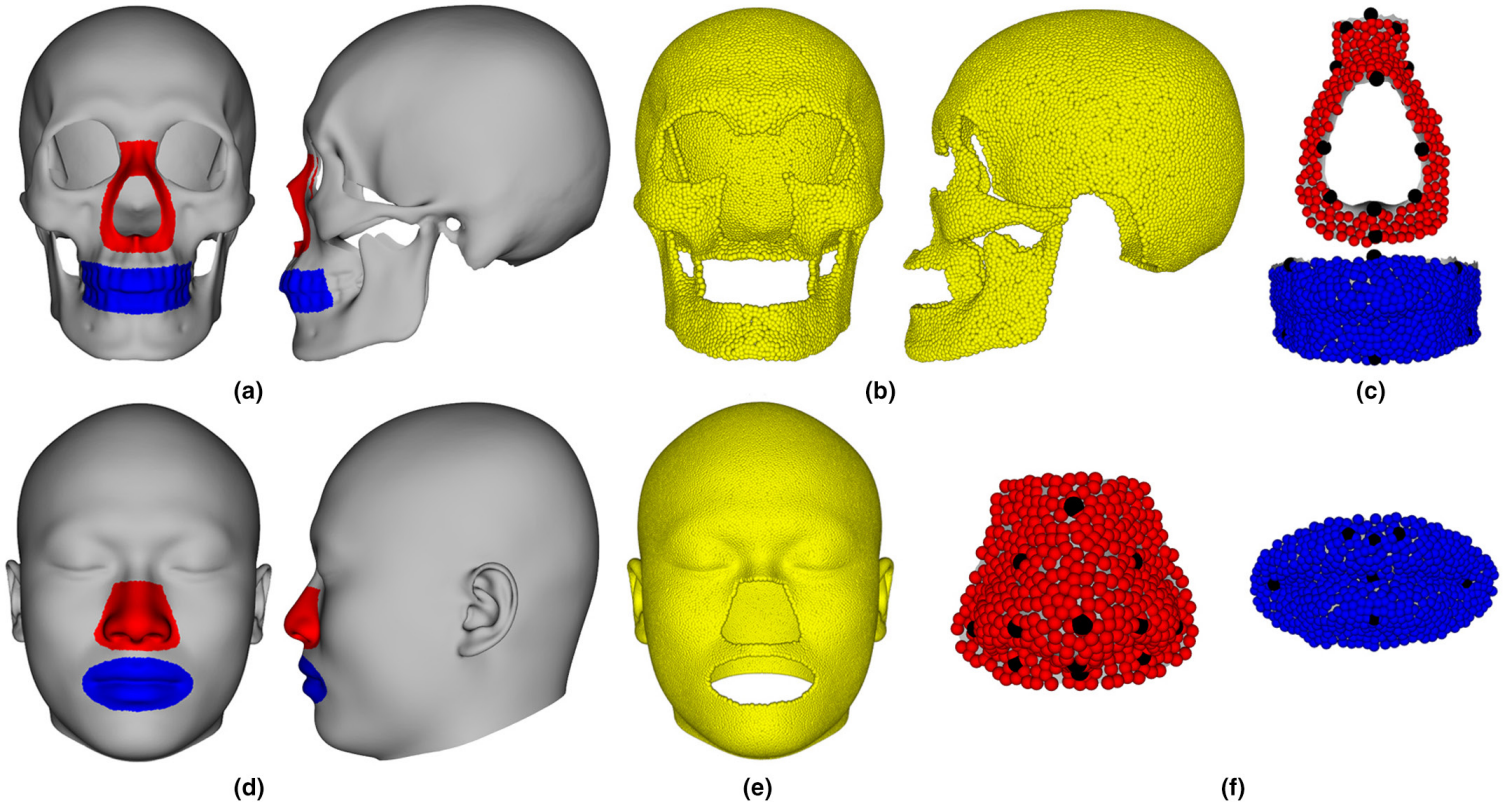
Template skull and face comprising the landmark and semilandmark configuration. (a) Skull envelope (gray), nasal (red), and oral (blue) hard tissue forms. (b) Skull envelope semilandmarks. (c) Landmarks (black), nasal (red), and oral (blue) semilandmarks. (d) Facial envelope (gray), nose (red), and mouth (blue) soft tissue forms. (e) Facial envelope semilandmarks. (f) Landmarks (black), nose (red), and mouth (blue) semilandmarks.

A total of 48 male adults aged 20–30 years were selected to explore craniofacial relationships and test performance of the proposed FA method in the present study. We manually separated the template skull into three components: the oral (blue), the nasal (red) hard tissues, and bony envelope (gray), and they are shown in Figure 1a. A total of 91 landmarks used in our previous study (Shui et al., 2021) were placed over the template skull and every subject skull. The Poisson‐disk algorithm (Corsini et al., 2012) was used to obtain 14,933 semilandmarks (Figure 1b) from the template bony envelope, excluding the temporal bone mastoid process and acoustic canal. As reported in our previous study (Shui et al., 2021), significant prediction bias around approximated ears, for example, discrete points that are far from the skull, can be avoided when average FSTDs are assigned to semilandmarks of the dry skull. Additionally, a total of 556 and 215 semilandmarks (Figure 1c) were sampled from the oral and nasal hard tissues of the template skull, respectively. We used the hybrid nonrigid registration approach combining TPS and nonrigid iterative closest point (NICP) (Amberg et al., 2007) methods to align the template skull to each skull with skull landmarks acting as constraints, and then projected semilandmarks of the deformed template skull onto each skull to yield high‐density semilandmarks.

We also established high‐density semilandmarks among faces. The template face was manually partitioned into three components: the mouth (blue), nose (red) soft tissues, and facial envelope (gray), as shown in Figure 1d. A total of 52 anatomical landmarks were chosen on the template face and every subject face (Table 1), where eight landmarks were located on the midline and 22 landmarks were bilaterally located. The Poisson‐disk sampling algorithm was used to obtain 150,000 (Figure 1e), 498 and 497 (Figure 1f) semilandmarks from each component of the template face, respectively. We used the hybrid nonrigid approach to warp the template face based on facial landmarks and then projected the semilandmarks of the deformed template face onto each subject face. The landmarks and high‐density semilandmarks are used to capture detailed morphologies of hard and soft tissues.

**TABLE 1:** Anatomical facial landmarks.

No.	Definition	Nature	Position
1 and 2	Medial canthus	Bilateral	Eyes
3 and 4	Lateral canthus	Bilateral	Eyes
5	Nasal bridge	Median	Nose
6	Middle of nose	Median	Nose
7	Tip of nose	Median	Nose
8	Subnasale	Median	Nose
9 and 10	External alar curvature	Bilateral	Nose
11 and 12	Superior alar curvature	Bilateral	Nose
13 and 14	Alare	Bilateral	Nose
15 and 16	Alar curvature point	Bilateral	Nose
17 and 18	Corner of mouth	Bilateral	Mouth
19 and 20	Crista philtra	Bilateral	Mouth
21	Middle of cupid's bow upper lip	Median	Mouth
22	Middle of oral fissure	Median	Mouth
23	Middle of bottom lip	Median	Mouth
24	Tip of chin	Median	Chin
25 and 26	Otobasion superius	Bilateral	Ears
27 and 28	Superior auricle	Bilateral	Ears
29 and 30	Posterior auricle	Bilateral	Ears
31 and 32	Inferior auricle	Bilateral	Ears
33 and 34	Anterior cymba concha	Bilateral	Ears
35 and 36	Superior cymba concha	Bilateral	Ears
37 and 38	Posterior concha	Bilateral	Ears
39 and 40	Intertragic incisure	Bilateral	Ears
41 and 42	Incisura intertragica	Bilateral	Ears
43 and 44	Tragion	Bilateral	Ears
45 and 46	Medial concha	Bilateral	Ears
47 and 48	Superior cavum concha	Bilateral	Ears
49 and 50	Otobasion posterius	Bilateral	Ears
51 and 52	Otobasion inferius	Bilateral	Ears

### 2.2 Methods

**FIGURE 2:**
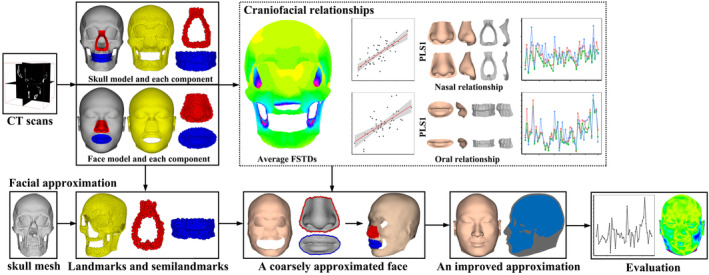
Workflow of quantifying craniofacial relationships and approximating facial appearances.

The proposed FA method includes two stages (Figure 2). In the first stage, we explored the craniofacial relationships of modern humans. We calculated average FSTDs at landmarks and high‐density semilandmarks of the skull envelope among samples to represent overall craniofacial relationships. The covariation of landmarks and semilandmarks between nasal hard and soft tissue shapes was explored using geometric morphometrics (GM), thereby approximating nose soft tissue shapes. We repeated the abovementioned step to explore the covariation between oral hard and soft tissue shapes and to generate mouth shapes. In the second stage, we presented a coarse‐to‐fine method to generate the facial appearance. The coarsely approximated face was generated by reassembling approximated facial envelope, nose, and mouth shapes. An improved approximated face was then generated by fitting the facial statistical shape model (SSM) to the coarse approximation, enabling the prediction of eye and ear shapes and filling in the missing geometry. We used resemblance comparison and recognition rate to evaluate the accuracy of the proposed method.

#### 2.2.1 Quantification of craniofacial relationships among human populations

##### Average FSTDs at landmarks and semilandmarks

The average FSTDs at landmarks and semilandmarks of the skull envelope were used to represent overall craniofacial relationships. FSTDs were defined as Euclidean distances between skull landmarks (or semilandmarks) and corresponding facial points. We used the normal vector (Toneva et al., 2021) of every landmark and semilandmark of the average skull as the measured orientation and then employed the same constant orientation to record FSTDs across samples. Furthermore, descriptive statistics of FSTDs among samples (e.g., mean and standard deviation) were calculated and a color map was used to show distributions of FSTDs.

Thereafter, average FSTDs at landmarks and semilandmarks were assigned to the external surface of the dry skull to obtain an approximated facial envelope, as follows:

(1) $$h_{i}=s_{i}+\vec{n_{i}}\cdot d_{i}$$

where $s_{i}\in {\mathbb{R}}^{3}$ represents *i*th landmark and semilandmark of the dry skull, and $h_{i}\in {\mathbb{R}}^{3}$ denotes the estimated point of facial envelope. $\vec{n_{i}}$ and $d_{i}$ represent the measured vector and average FSTDs. These facial points were then converted to a set of triangle meshes by the ball‐pivoting algorithm (Bernardini et al., 1999).

##### Quantification of relationships between nasal (or oral) hard and soft tissue shapes

To explore relationships between nasal (or oral) hard and soft tissue shapes, a generalized Procrustes analysis (GPA) algorithm (Mitteroecker & Gunz, 2009) was applied to the landmark and semilandmark configurations of hard and soft tissues across all individuals separately. Subsequently, a two‐block partial least squares (2B‐PLS) analysis (Rohlf & Corti, 2000) was used to explain the covariation between hard and soft tissues of the nose and mouth using the R package “geomorph”(Adams & Otárola‐Castillo, 2013), with Block‐1 defined here as the hard tissue shapes and Block‐2 as the soft tissue shapes. Unlike principal component analysis (PCA), 2B‐PLS maximizes covariance patterns, as derived from the cross‐covariance matrix, to produce pairs of component axes. RV coefficients (Robert & Escoufier, 1976) were used to measure overall associations between the two matrices as generated using PLS scores derived from two different configurations by qualifying the multivariate extension of the correlation coefficient. A permutation test with 1000 runs was performed to assess the significance of differences. To examine the morphological variations of shapes representing the extreme limits of PLS, fitted landmarks and semilandmarks along the positive (+) and negative (−) limits of PLS 1 were generated and then fitted shapes were generated by warping the mean shape using TPS. Regional variations of two different fitted shapes were found by observing movement patterns of landmarks and semilandmarks.

The next step was to approximate the nose (or mouth) soft tissue shapes of the dry skull. To reduce data dimensionality, PCA was applied to Procrustes shape coordinates of hard and soft tissues of every sample onto the shape space separately. A linear multiple regression was used to quantify relationships between the PC scores of hard (independent variable) and soft (dependent variable) tissue shapes as follows:

(2) $$M_{\text{organ}}=\arg\min{\left\|{\mathrm{n\beta}}_{h}\cdot M_{\text{organ}}-{\mathrm{n\beta}}_{s}\right\|}^{2}+{\eta_{\text{organ}}}^{2}{\left\|M_{\text{organ}}\right\|}^{2}$$

where ${\mathrm{n\beta}}_{h}$ and ${\mathrm{n\beta}}_{s}$ represent PC scores of nasal (or oral) hard and soft tissues, respectively, and $M_{\text{organ}}$ denotes the craniofacial relationships. $\eta_{\text{organ}}$ denotes the weighting coefficient that is related to the number and standard deviation of PCs.

Based on the learned relationships and the calculated PC scores derived from landmark and semilandmarks of hard tissue, coefficients of approximated nose (or mouth) soft tissue shapes were computed. Landmarks and semilandmarks of approximated soft tissues were obtained by adding the mean nose (or mouth) shape among samples to the linear combination of the computed coefficients and PCs.

Here we used leave‐one‐out cross‐validation (LOOCV), that is, one sample within the dataset was selected for use as test data, while the other samples served as training data, to validate the accuracy of the approximated results. This process was then repeated with each sample used once as the test data. Procrustes distances between approximated and actual shapes were calculated to quantify gross differences. To contextualize the extent to which Procrustes distances between approximated and actual shapes differed, the ratio of Procrustes distance divided by the average distance between individuals and mean shape was further calculated. Additionally, nose (or mouth) soft tissue shapes were generated by deforming the mean shape using TPS. Geometric differences in landmarks and semilandmarks between approximated and actual nose (or mouth) shapes were transferred to the shapes to generate a color map for use in comparing regional shape differences.

#### 2.2.2 Facial approximation

##### A coarsely approximated face

**FIGURE 3:**
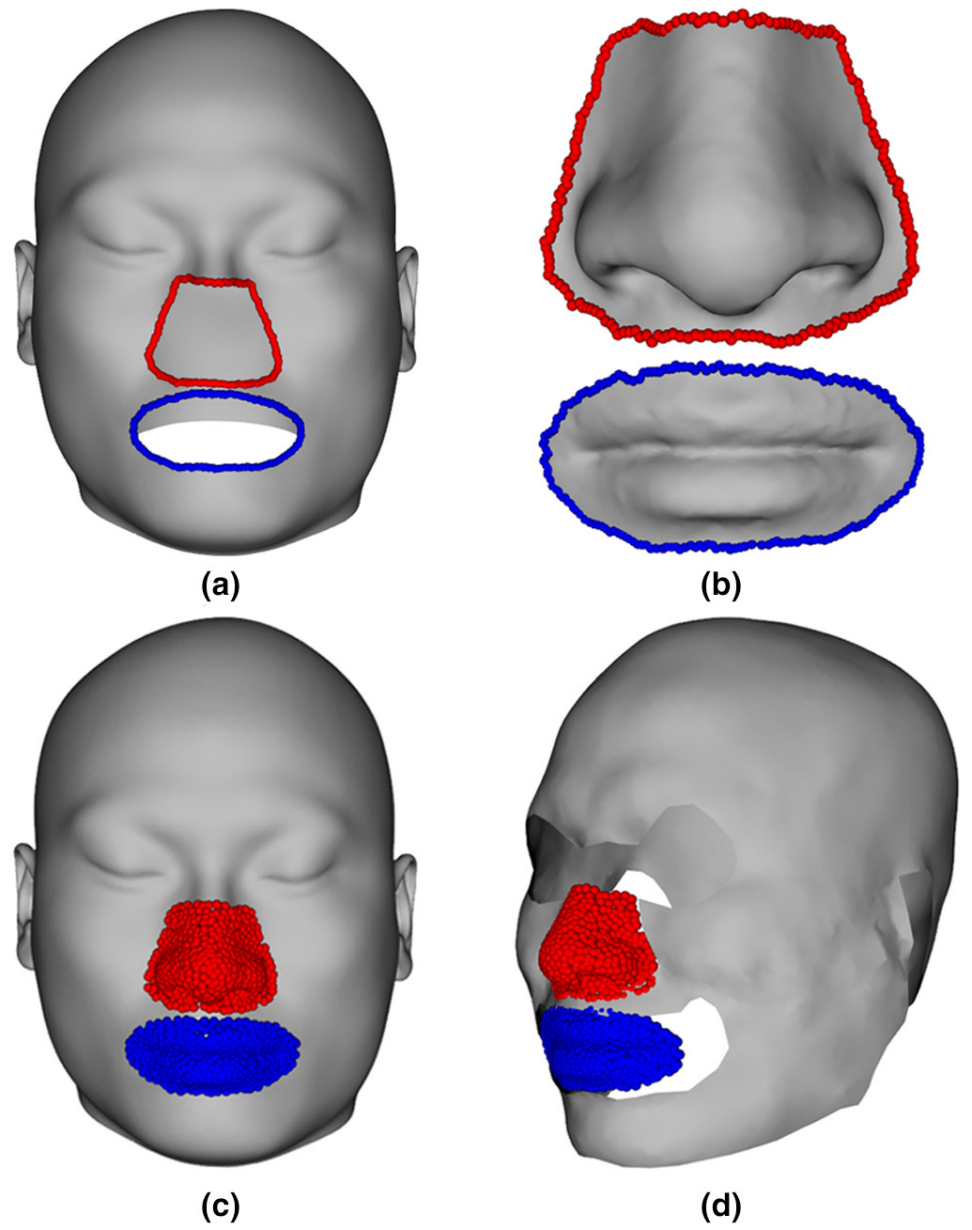
The coarsely approximated face. (a) Deformed average facial envelope with boundary curves. (b) Approximated nose and mouth shapes with boundary curves. (c) The superimposition of deformed average facial envelope (gray) and transformed approximated nose (red) and mouth (blue) soft tissues. (d) The coarsely approximated face comprising approximated facial envelope (gray), and the transformed nose (red) and mouth (blue) soft tissues.

Once landmarks and semilandmarks of the dry skull were generated, we were able to recreate the facial envelope based on average FSTDs (Equation 1) and approximate the probable nose and mouth shapes based on the learned relationships (Equation 2), respectively. However, they have different locations, orientations, and sizes. A critical step involved the calculation of the transformation (translation, rotation, and scaling) of approximated nose and mouth soft tissue shapes to fit them onto the approximated facial envelope, thereby generating the coarsely approximated face (Figure 3). We used a deformation‐based approach to warp the average facial envelope followed by transformation to align the average skull with the dry skull (Figure 3a). The boundary curves of the deformed facial envelope with approximated nose (red) and mouth (blue) shapes were extracted (Figure 3b). The least‐squares algorithm was conducted to register approximated soft tissues of each component (Figure 3c,d) by minimizing the sum of squared Euclidean distances between boundary curves as follows:

(3) $$\underset{R,\vec{t},S}{\arg\min}\sum_{i=1}^{m}{\left\|q_{i}-\left(R\cdot S\cdot p_{i}+\vec{t}\right)\right\|}^{2}$$

where $p_{i}\in {\mathbb{R}}^{3}$ and $q_{i}\in {\mathbb{R}}^{3}$ represent corresponding boundary vertices of the facial envelope and nose (or mouth) shapes, *m* denotes the number of correspondences and $\text{R}\in \mathrm{SO}\left(3\right)$, $\vec{t}=\left(t_{x},t_{y},t_{z}\right)$ and S represent rotation, translation, and uniform scaling matrices, respectively.

##### An improved approximated face

The improved approximated face was recreated by fitting the facial SSM to the coarsely approximated face.

Facial SSM. Generalized Procrustes analysis was applied to facial landmarks and semilandmarks among samples to generate Procrustes shape coordinates, and then PCA was used to construct the facial SSM, which represents the probability distribution of faces as a prior knowledge (Brunton et al., 2014). Thus, every face within the dataset can be represented as follows:

(4) $$F\left(\alpha \right)=\vec{F}+\sum_{i=1}^{d}\mu_{i}\alpha_{i}$$

where $F\left(\alpha \right)$ represents an arbitrary individual in the sample, $\vec{F}={\left[x_{1},y_{1},z_{1},\cdots ,x_{n},y_{n},z_{n}\right]}^{T}$ denotes the average face, and *n* denotes the number of landmarks and semilandmarks. $\alpha =\left(\alpha_{1},\alpha_{2},\cdots ,\alpha_{d}\right)$ and $U=\left(\mu_{1},\mu_{2},\cdots ,\mu_{d}\right)\in {\mathbb{R}}^{3n\times d}$ represent PC scores and corresponding orthogonal PCs derived from the covariance matrix, and *d* represents the number of PCs as determined from the cumulative proportion.

Facial fitting. Since the approximated facial envelope and $\vec{F}$ were located in different coordinate systems, we transformed the approximated facial envelope to fit $\vec{F}$ based on point correspondences. These correspondences were established by computing intersection points from each placed point on the skull to facial envelope and deformed average facial envelope (Shui et al., 2021). Moreover, we recognized the index of every intersection point of deformed average face, thereby considering the intersection points of facial envelope and selected points of every individual with the same indices serving as point correspondences. Afterward, the coefficients were optimized to fit the coarsely approximated face as follows:

(5) $$\underset{\beta }{\arg\min}\left({\left\|U_{s}\cdot \beta -\left({\mathrm{tp}}_{i}-\vec{F_{i}}\right)\right\|}^{2}+\lambda^{2}{\left\|\beta \right\|}^{2}\right)$$

where $\beta =\left(b_{1},b_{2},\cdots b_{d}\right)$ represents coefficients of the fitting approximation in the facial SSM. ${\mathrm{tp}}_{i}$ and $\vec{F_{i}}$ represent the *i*th correspondence between the transformed facial envelope and average face of SSM. $U_{s}\subset U$ represents the subset of PCs of SSM and λ denotes the weighting coefficient.

Then, the fitting approximation was determined as follows:

(6) $$Q\left(\beta \right)=\vec{F}+\sum_{i=1}^{d}\mu_{i}b_{i}$$

To minimize fitting errors and utilize the originally approximated nose and mouth soft tissues, Laplacian deformation (Sorkine et al., 2004) was used to deform $Q\left(\beta \right)$ to the coarsely approximated soft tissues. The established point correspondences over the facial envelope and the approximated landmarks and semilandmarks of nose and mouth soft tissues were regarded as fixed constant anchors to constrain the deformation. Hence an improved approximated face was generated as follows:

(7) $$E=\sum_{i=1}^{n}{\left\|\delta_{i}\left(g_{i}\right)-\delta_{i}\left(v_{i}\right)\right\|}^{2}+\sum_{j=1}^{m}{\left\|l_{j}-v_{j}\right\|}^{2}$$

where $g_{i}\in {\mathbb{R}}^{3}$ and $v_{i}\in {\mathbb{R}}^{3}$ represent the *i*‐th vertex of improved face and fitting approximation, and $\delta_{i}$ represents Laplacian coordinates of every vertex derived from one‐ring adjacent vertices. $l_{j}$ and $v_{j}$ represent the *j*‐th correspondence of the coarsely approximated face and fitting approximation.

##### Assessment of the approximated result

LOOCV was performed repeatedly with each sample used once as the test data and other samples as the training data. We used the resemblance comparison (i.e., geometric difference between approximated and actual faces) to test the accuracy. A total of 16 landmarks and 59 semilandmarks (Smith et al., 2020) were placed over the approximated and actual faces. Procrustes superimposition (Gunz & Mitteroecker, 2013) was used to align them and Procrustes distances were calculated to quantify gross surface difference. Additionally, a geometric difference was calculated by computing the average Euclidean distance between dense point correspondences established by searching the nearest point between these two faces. Small differences would indicate that the approximated face bears a strong resemblance to the actual one, whereas large differences would reflect dissimilarity. A color map of Euclidean distances between correspondences of the approximated and actual faces was used to recognize the regional surface difference.

A recognition rate (i.e., a comparison of an approximated face to every face within the face pool comprising every actual face) was used to evaluate the accuracy (Stephan & Henneberg, 2006). A correct match indicates that the approximated face is one of the *k* most resembled faces regarding the actual face through a comparison of the Procrustes distance, called top‐k rank. The recognition rate (%) was calculated as the percentage of the number of correct matches divided by the number of samples.

## 3 RESULTS

We analyzed craniofacial relationships among modern humans and then tested the accuracy of the proposed FA method by comparing the approximated faces with actual ones.

### 3.1 Quantification of craniofacial relationships among modern humans

#### 3.1.1 FSTDs at landmarks and semilandmarks of modern humans

**FIGURE 4:**
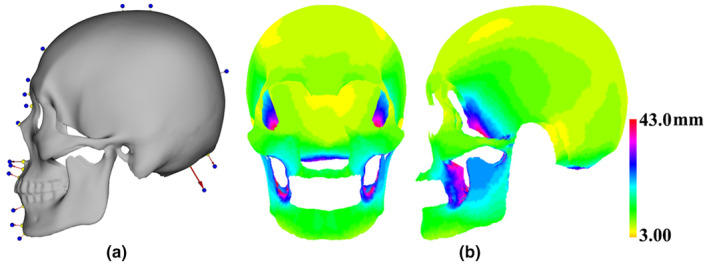
Average FSTDs at landmarks and semilandmarks. (a) Measured orientations (red arrows) of landmarks (yellow) located within the mid‐sagittal plane and intersection points (blue) on the face. (b) A color map of average FSTDs.

Measured orientations of landmarks located within the mid‐sagittal plane were illustrated and the FSTDs of each individual were calculated by computing the Euclidean distance between landmarks and semilandmarks of hard tissues and corresponding intersection points on the face (Figure 4a). Average FSTDs at landmarks and semilandmarks across samples were visualized using a color map (Figure 4b). The average value of FSTDs was almost 9.29 mm with a standard deviation of almost 6.48 mm. Our results indicated that FSTDs were distributed almost symmetrically with regard to the mid‐sagittal plane. The smallest FSTDs were observed around the frontal bone and cranial vault, while the largest FSTDs were found around the lateral maxillary bone and mandible (corresponding to cheek tissue), the greater wing of the sphenoid bone and the base of the occipital bone.

#### 3.1.2 Quantification of the nose relationship

To explore associations between nasal hard and soft tissue shapes across samples (*n* = 48), we performed 2B‐PLS analysis of two different configurations of nasal bone versus both landmarks and semilandmarks of soft tissues. A summary of PLS correlation coefficients is presented in Table 2. Because only the first three PLS dimensions accounted for more than 10% of the total shape variance, RV coefficients of these three PLS dimensions and all dimensions were calculated. The coefficient of all dimensions for landmarks alone of hard tissues (RV = 0.297) and that for landmarks and semilandmarks together (RV = 0.383) reveals significant but comparatively weak covariation between nasal hard and soft tissues. The use of semilandmarks of hard tissues increases RV correlation, while an increase in the number of PLS dimensions reduces the strength of RV correlation.

**TABLE 2:** 2B‐PLS analysis between nasal hard and soft tissues.

Configurations of hard tissues	PLS axes				RV (the first three PLS)		RV (all the PLS)	
	PLS	Correlation	*p* value	%	Correlation	*p* value	Correlation	*p* value
Landmarks	PLS1	0.748	<0.01	32.84%	0.389	<0.01	0.297	<0.01
	PLS2	0.599	<0.01	20.20%				
	PLS3	0.634	<0.01	15.37%				
Landmarks and semilandmarks	PLS1	0.753	<0.01	34.69%	0.525	<0.01	0.383	<0.01
	PLS2	0.793	<0.01	25.41%				
	PLS3	0.645	<0.01	13.25%				

**FIGURE 5:**
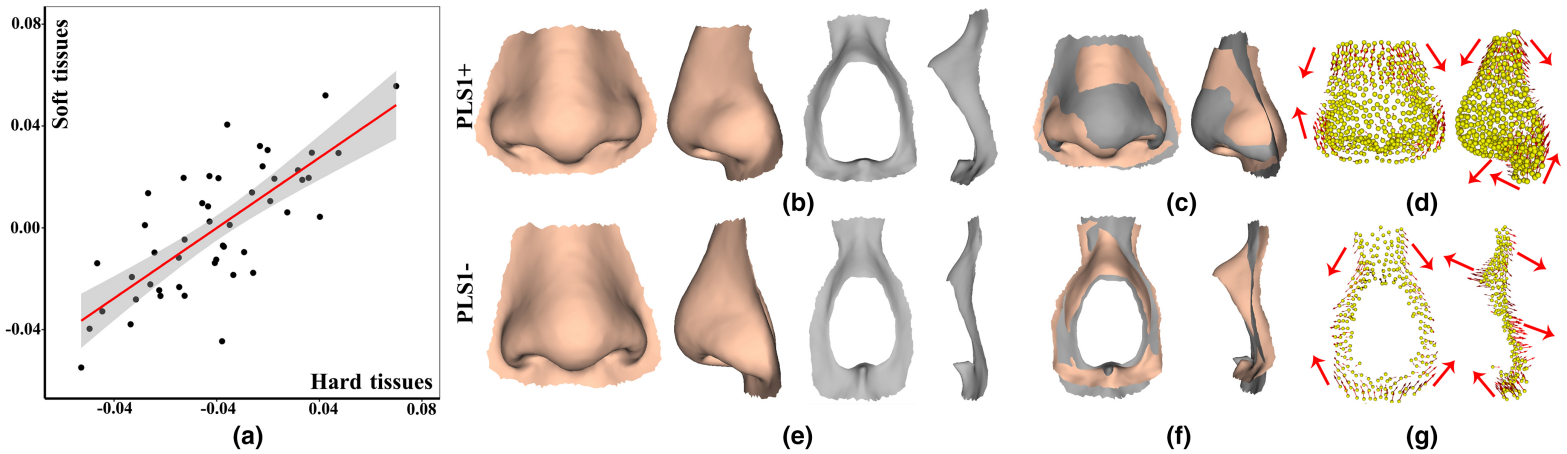
PLS1 covariation of landmarks and semilandmarks between nasal hard and soft tissues. (a) Scatterplots of PLS1 axes of nasal hard and soft tissues. (b) Fitted shapes (PLS1+) along the positive axis. (c) Superimposition of PLS1+ (peach) and PLS1− (gray) of nose soft tissues. (d) Movement patterns of soft tissues. (e) Fitted shapes (PLS1−) along the negative axis. (f) Superimposition of PLS1+ (peach) and PLS1− (gray) of nasal hard tissues. (g) Movement patterns of hard tissues.

Given the small amount of covariation explained by subsequent PLS dimensions and the stronger RV correlation derived from landmarks and semilandmarks of hard tissues, the result of PLS1 was further analyzed. Figure 5 provides a bivariate plot of PLS1 for nasal hard tissues (horizontal axis) and soft tissues (vertical axis) with fitted shapes depicting morphological changes. The morphological pattern of covariation explained by PLS1 is highly significant (*r* = 0.753) and the red line represents the linearly fitted regression of soft tissues (dependent variables) onto hard tissues (independent variables) with confidence intervals (Figure 5a). The fitted hard and soft tissue shapes with positive (PLS1+) and negative (PLS1‐) axis are illustrated (Figure 5b,e). As evidenced by the superimposition of fitted shapes (Figure 5c,f) and movement patterns (red arrows) of landmarks and semilandmarks with PLS1‐ serving as the reference (Figure 5d,g), the covariation detected in the PLS1 shows nose soft tissues changes seem to be correlate with those of the hard tissues, for example, the right‐to‐left width and superior‐to‐inferior height of the nasal bone covary with those of the soft tissues. Specifically, nose soft tissues tend to widen and shorten with increasing PLS1 score, whereby PLS1+ exhibits a relatively shorter, wider, and more protruding rhinion and bridge, a wider and bigger nasal alar, a slightly protruding infratip lobule and columella, and a more protruding subnasale compared to PLS1−. Additionally, as the PLS score becomes increasingly positive, nasal hard tissues tend to widen, shorter, and protrude, while the region around the nasal spine becomes smaller and more contracted.

**FIGURE 6:**
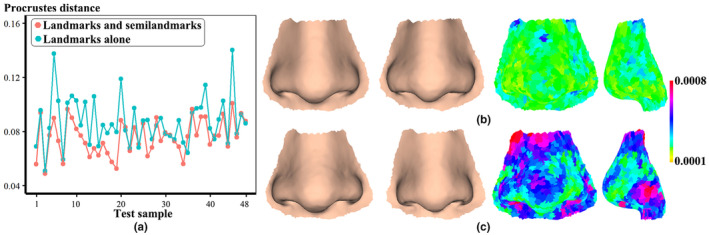
Geometric discrepancy of approximated and actual nose soft tissues. (a) Geometric discrepancy of each test sample was derived using two different configurations of hard tissues. (b) Comparisons of approximated (left) and actual (second left) nose soft tissues with the minimum Procrustes distance. (c) Comparison of approximated (left) and actual (second left) nose soft tissues with the maximum Procrustes distance.

Regarding the scatterplots (Figure 6a) of Procrustes distance (vertical axis) between approximated and actual soft tissues of each test sample (horizontal axis) and a summary of descriptive statistics (Table 3), for example, mean, standard deviation, maximum ratio (*MaxR*), and minimum ratio (*MinR*), a smaller mean geometric deviation (0.0761 ± 0.0131) suggests that the use of landmarks and semilandmarks of hard tissues is superior to using landmarks alone. However, a large ratio variation (*MaxR* = 1.4627) indicates that the approximated nose has a greater error. The approximation biases derived from landmarks and semilandmarks of hard tissues are further analyzed. A color map (right of figure) of geometric difference shows the approximated nose (left) bears the greatest resemblance to the actual one (second left), as reflected by the minimum Procrustes distance (Figure 6b). The approximation with the maximum prediction errors indicate that the greatest differences are observed around the nasal alar and tip, rhinion, the bridge, and subnasale (Figure 6c).

**TABLE 3:** A comparison of the approximated and actual nose soft tissues.

Configurations of hard tissues	Geometric deviation			
	Mean	SD	*MaxR*	*MinR*
Landmarks and semilandmarks	0.0761	0.0131	1.4627	0.7079
Landmarks	0.0874	0.0179	2.0323	0.7390

#### 3.1.3 Quantification of the mouth relationship

2B‐PLS analysis was used to explore covariations of oral hard and soft tissues across samples (*n* = 48). A summary of PLS correlations and RV coefficients obtained using two different configurations of oral structure is presented in Table 4. Due to the fact that only the first two PLS dimensions explained more than 10% of the total shape variance, RV coefficients of these PLS dimensions alone, and also all dimensions together, were calculated, with this analysis revealing that the weak correlation exists between the two blocks. Additionally, each PLS and RV correlation derived from landmark and semilandmark configurations of hard tissue is greater than those derived from landmarks alone, while an increase in the number of dimensions leads to reduced RV correlation.

**TABLE 4:** 2B‐PLS analysis between oral hard and soft tissues.

Configurations of hard tissues	PLS axes				RV (the first two PLS)		RV (all the PLS)	
	PLS	Correlation	*p value*	%	Correlation	*p value*	Correlation	*p value*
Landmark	PLS1	0.633	<0.01	63.83%	0.374	<0.01	0.271	<0.01
	PLS2	0.704	<0.01	12.91%				
Landmarks and semilandmarks	PLS1	0.661	<0.01	56.55%	0.412	<0.01	0.325	<0.01
	PLS2	0.768	<0.01	21.16%				

**FIGURE 7:**
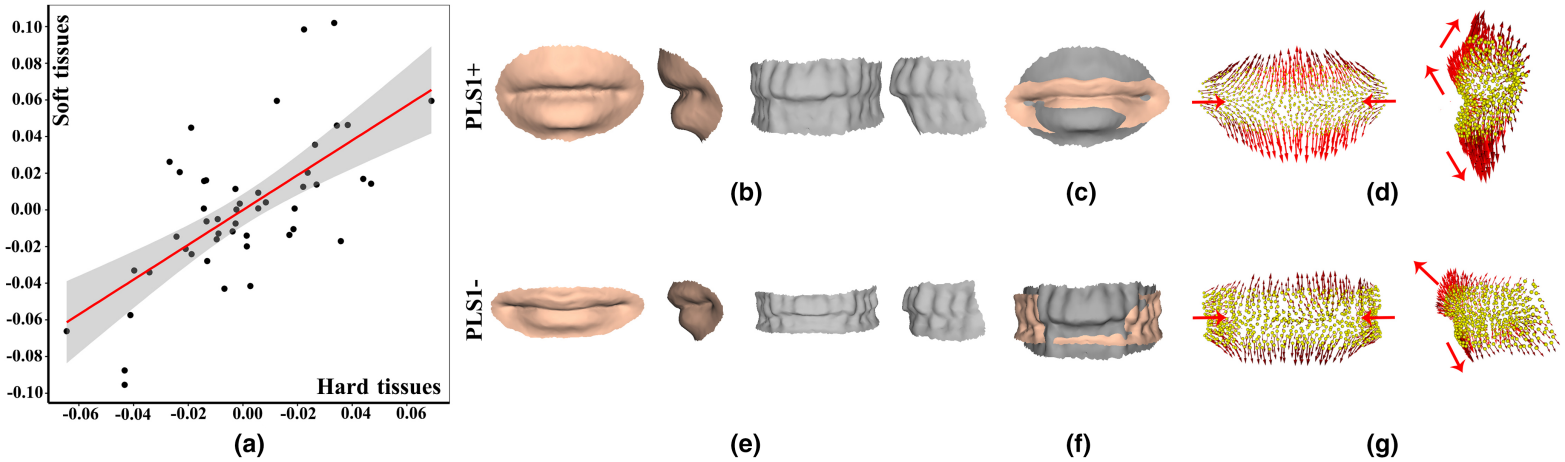
PLS1 covariation between oral hard and soft tissue shapes. (a) Scatterplots of PLS1 axes of landmarks and semilandmarks of hard and soft tissues. (b) Fitted shapes (PLS1+) along the positive axis. (c) Superimposition of PLS1+ (peach) and PLS1− (gray) of mouth soft tissues. (d) Movement patterns (red row) of soft tissues. (e) Fitted shapes (PLS1−) along the negative axis. (f) Superimposition of PLS1+ (peach) and PLS1− (gray) of oral hard tissues. (g) Movement patterns (red row) of hard tissues.

The result of PLS1 derived from landmarks and semilandmarks was further analyzed. Figure 7 shows scatterplots of PLS1 for hard tissues (horizontal axis) and soft tissues (vertical axis). The morphological pattern of covariation explained by PLS1 is highly significant (*r* = 0.661). The fitted hard and soft tissue shapes with positive (PLS1+) and negative (PLS1−) axis are illustrated (Figure 7b,e). As evidenced by superimposition of the fitted shapes (Figure 7c,f) and movement patterns (red arrows) of landmarks and semilandmarks with PLS1− serving as the reference (Figure 7d,g), PLS1 exhibits a pattern of covariance such that right‐to‐left width, superior‐to‐inferior height and the protruding shapes of oral hard tissues covary with soft tissues. Mouth soft tissues tend to narrow and enlarge with an increasing PLS score, and there is an increasing PLS1+ associated with narrowing and protruding upper and lower lips, together with an increasingly protruding and enlarged open mouth and an oral fissure located on a more backward position. Meanwhile, an increasingly positive PLS score is associated with increasingly narrow and enlarged oral hard tissue shapes and increasingly protruding anterior teeth.

**FIGURE 8:**
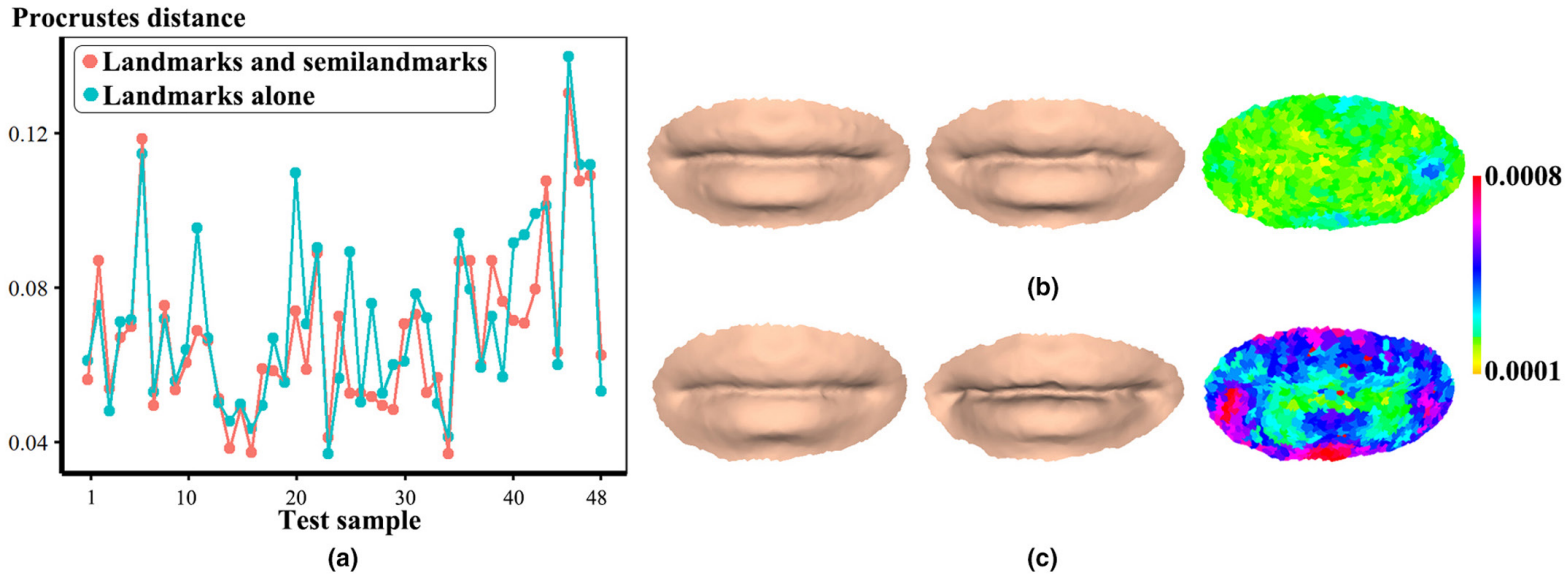
Geometric discrepancy of the approximated and actual mouth soft tissues. (a) Geometric discrepancy of each test sample was derived using two different configurations. (b) Comparison of approximated (left) and actual (middle) mouth soft tissues with the minimum Procrustes distance. (c) Comparison of approximated (left) and actual (middle) mouth soft tissues with the maximum Procrustes distance.

Regarding the scatterplots (Figure 8a) of Procrustes distance (vertical axis) between approximated and actual soft tissues of each test sample (horizontal axis), 62.5% (*n* = 30) of test samples using landmarks and semilandmarks of hard tissues yielded smaller Procrustes distances as compared to results derived from landmarks alone. A summary of descriptive statistics of Procrustes distance is presented in Table 5. A smaller mean geometric deviation (0.0684 ± 0.0221) indicates that the use of landmarks and semilandmarks probably contributes to improving approximation accuracy. However, a large ratio variation (*MaxR* = 2.0157) shows that the approximated mouth shapes have a greater error. A color map (right of figure) of geometric difference exhibits that the approximated mouth (left) bears the greatest resemblance to the actual one (middle), as reflected by the minimum Procrustes distance (Figure 8b). The approximation with the maximum prediction errors indicates that the greatest changes are found around boundary regions, as well as the philtrum, mouth corners, the cupid's bow, and lower lip (Figure 8c).

**TABLE 5:** Comparison of approximated and actual mouth soft tissues.

Configurations of hard tissues	Geometric deviation			
	Mean	SD	*MaxR*	*MinR*
Landmarks and semilandmarks	0.0684	0.0221	2.0157	0.5731
Landmarks	0.0714	0.0229	2.1619	0.5715

### 3.2 Evaluation of the proposed FA method

**FIGURE 9:**
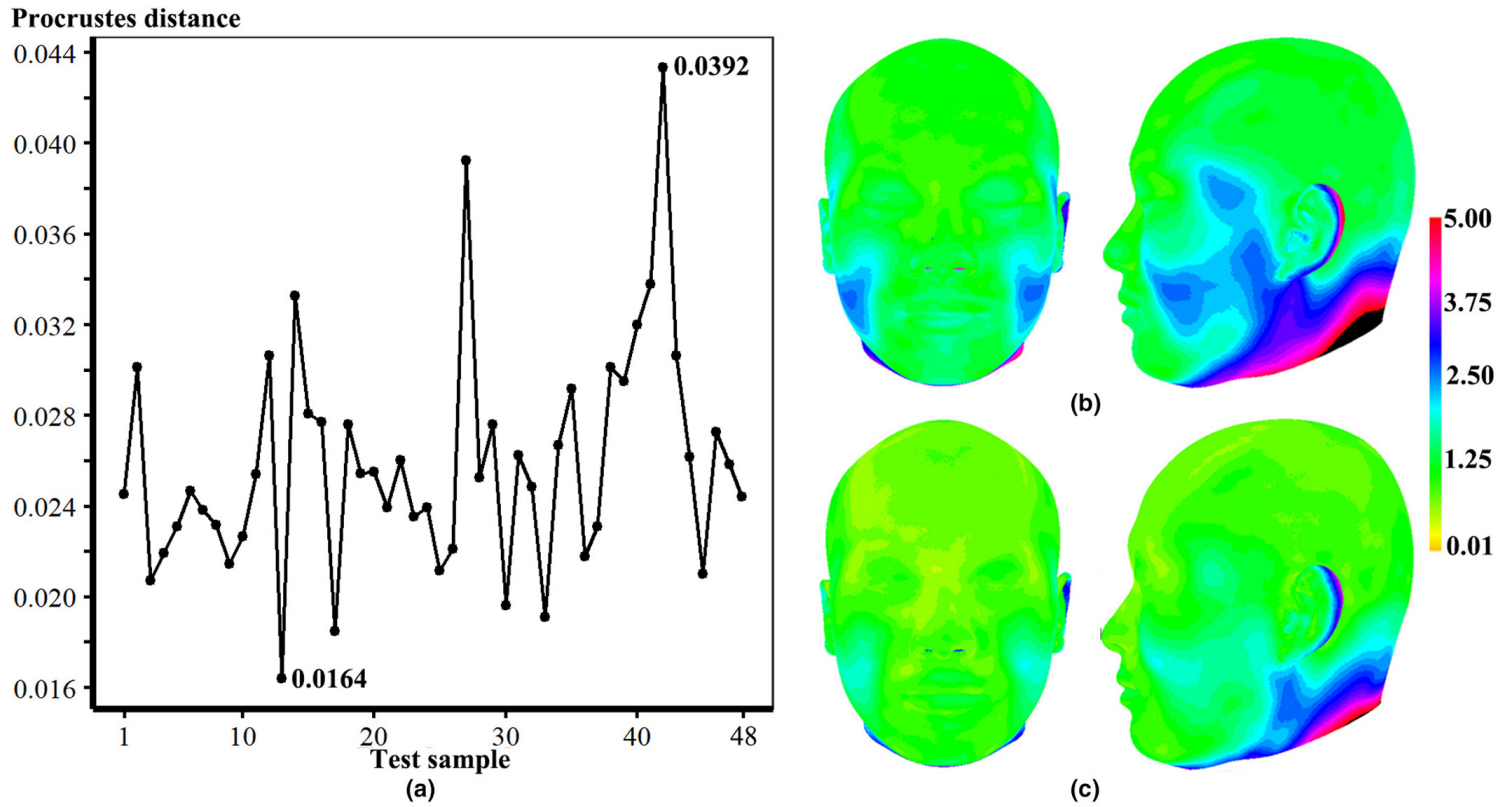
A comparison of the approximated and actual faces. (a) Procrustes distance between approximated and actual faces of each test sample. (b) Average approximation deviations. (c) Standard deviation of approximation deviations.

**FIGURE 10:**
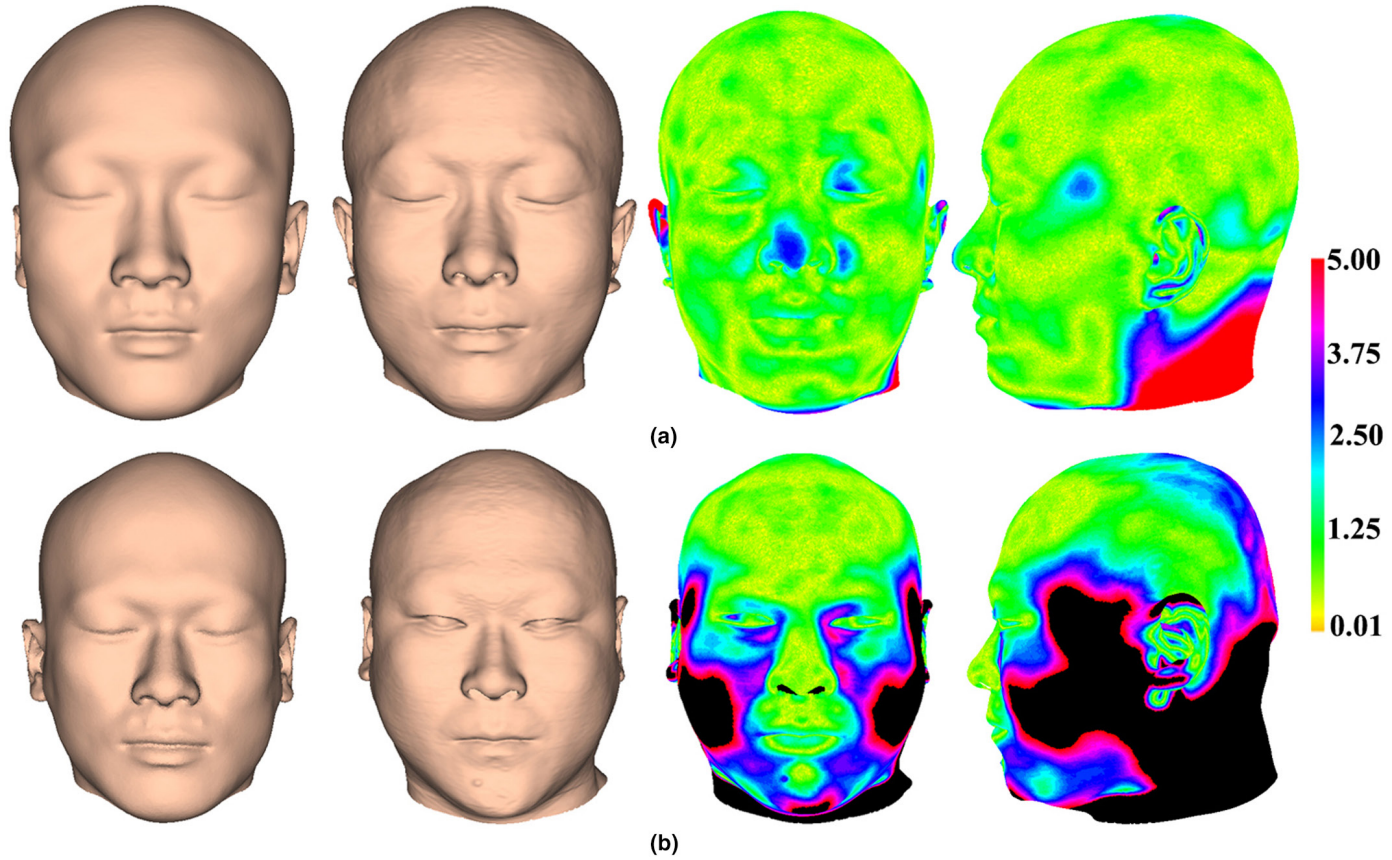
The approximated and actual faces of two test samples. (a) The approximated face with the smallest deviation. (b) The approximated face with the greatest deviation.

**FIGURE 11:**
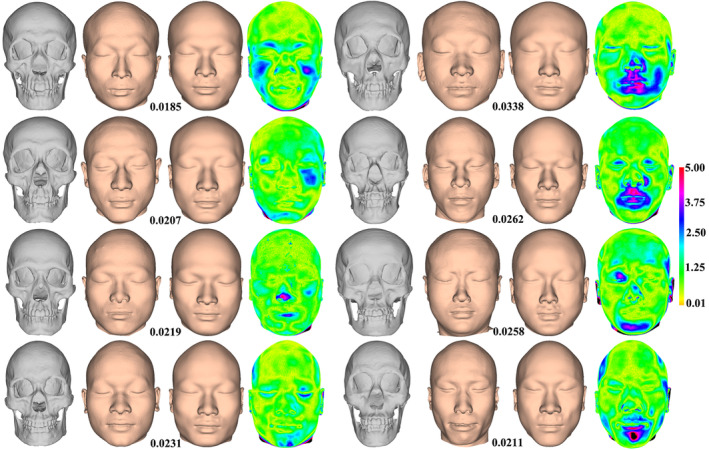
The approximated and actual faces of eight test samples. Each example includes the test skull (gray), actual (second column), and approximated (third column) faces, and a color map of geometric deviation (rightmost).

We test performance of the FA method by computing the Procrustes distance (vertical axis) between approximated and actual faces of test samples (horizontal axis), yielding an average Procrustes distance of 0.0258 (Figure 9a). Moreover, the average Euclidean distance between approximated and actual faces is 1.79 mm and 72.92% (*n* = 35) of samples show a geometric difference of less than 2.0 mm. To contextualize the extent of approximation bias, the mean (Figure 9b) and standard deviation (Figure 9c) of the geometric difference among test samples are illustrated. The results show that the greatest differences are found around the cheeks, the lateral temporal‐cheek, the ears, and the back of head and neck. A color map of geometric difference shows the approximated face (left of figure) closely resembles the actual one (second left), as reflected by the minimum Procrustes distance (0.0164) with an average Euclidean distance of 1.12 mm (Figure 10a). Figure 10b shows the approximated face corresponding to the maximum Procrustes distance (0.0392), in which the average Euclidean distance is 3.28 mm. The greatest shape differences are observed around the cheeks, lateral temporal‐cheek, nasolabial folds, medial inferior orbital, the chin, lateral forehead, ears, and the back of head and neck. Figure 11 shows the approximated faces of another eight test examples with the Procrustes distance between approximated and actual faces.

Additionally, we calculated the Procrustes distance between the approximated face and every face within the face pool. The top‐1 recognition rate of 91.67% (*n* = 44) and the top‐3 recognition rate of 95.83% (*n* = 46) demonstrate the effectiveness of the proposed method.

### 3.3 Facial approximation of the upper cave (UC) 101 skull

**FIGURE 12:**
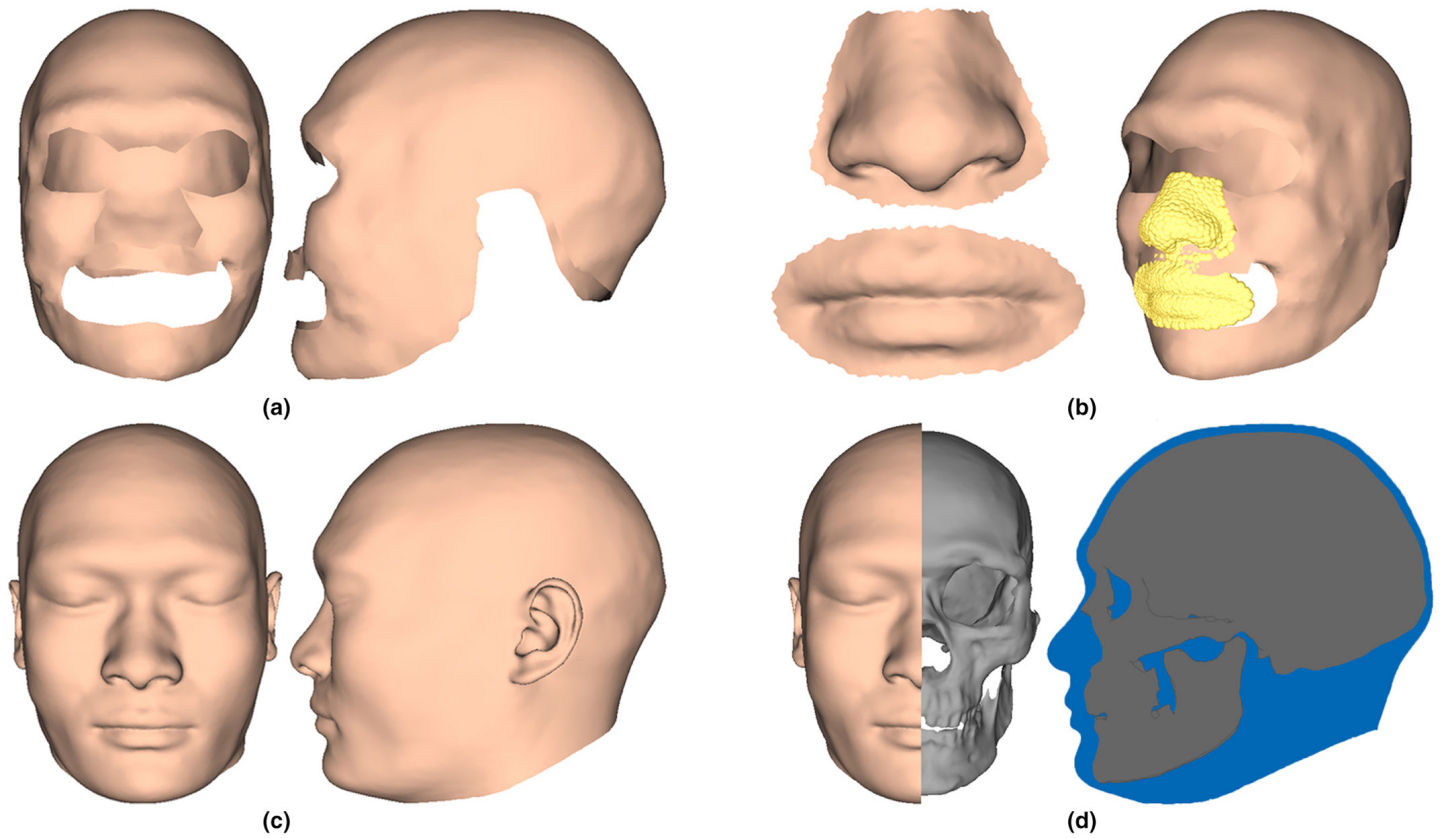
Facial approximation of UC 101. (a) The approximated facial envelope. (b) Alignment of the approximated facial envelope, mouth, and nose. (c) Improved approximated face. (d) Superimposition of the approximated face and UC 101.

The UC 101 skull, which dates back to almost 30,000 years BP, was discovered in the upper cave of Zhoukoudian in northern China in 1930. It exhibits a longer and lower cranial vault, a broader forehead, a more pronounced superciliary arch and a higher nasal bridge compared with the corresponding features of modern humans. We approximate the facial appearance of UC 101 using the proposed method. The facial envelope (Figure 12a), nose and mouth soft tissues (Figure 12b) are approximated based on the learned craniofacial relationships and these results are then reassembled to yield the coarsely approximated face. However, it still lacks eyes, ears, and missing geometry. Figure 12c shows the improved approximated face by fitting the facial SSM. As compared to modern human faces, the approximated face exhibits an elongated shape, a sloped forehead, stronger, and wider eyebrows and a wider nose bridge. The superimposition of the approximated face and UC 101 (Figure 12d) indicates that the main patterns and profile of the approximated face are almost consistent with those of UC 101.

## 4 DISCUSSION

Despite recent progress in improving the accuracy of the approximated face using computerized methods, existing methods suffer from several issues, including a limited understanding of detailed craniofacial relationships and challenges related to the assignment of the learned relationships to obtain a reliable and accurate facial appearance. This study presents a computerized method for quantifying the overall craniofacial relationships and the covariations between hard and soft tissues of both the nose and mouth. Furthermore, we applied the learned relationships to approximate the facial envelope, nose, and mouth soft tissues and afterward employed facial SSM to enhance the accuracy of approximated results. The smaller resemblance comparison and greater recognition rate demonstrate the effectiveness of the proposed FA method.

### 4.1 The quantification of craniofacial relationships

As FSTDs at landmarks cannot provide sufficient information, recent studies employed FSTDs at landmarks and high‐density semilandmarks to intuitively explain the detailed craniofacial relationships (Gietzen et al., 2019; Shui et al., 2016). Notably, they are influenced by the choice of algorithm to establish the semilandmarks among skulls, and dependent upon determination of measured orientation to record FSTDs. In morphometrics, sliding approaches by minimizing Procrustes distance or bending energy (Gunz & Mitteroecker, 2013) have been developed to project semilandmarks of the template to each specimen based on equivalent landmarks. The present study used the hybrid nonrigid registration approach which can yield consistent morphometric results (Shui et al., 2023) to identify high‐density semilandmarks. Furthermore, the constant orientation, that is, normal vector of every landmark and semilandmark of the average skull, is treated as the measured direction across samples and can approximate dense points on the facial envelope. Compared to FSTDs computed by using cylindrical vectors (Shui et al., 2016) and searching the nearest points from faces (Gietzen et al., 2019), this avoids the use of inconsistent directions in FSTD measurements between samples and reduces the generation of incorrect facial points (Shui et al., 2021).

The nose is one of the most prominent features of the human face, and hence its approximated morphology is capable of enhancing the realism of the approximated face and facial identification. The quantification of covariations between hard and soft tissues provides reliable evidence and anatomical guidance for the approximated nose soft tissues. The correlations of anthropomorphic measurements computed by landmarks are commonly used in the conventional method and then regression equations are obtained to predict probable position and measurement of soft tissues (Lee et al., 2014; Rynn & Wilkinson, 2006). For example, a recent study found basis nasi and nasospinale protrusions have the strongest correlation (*r* = 0.499) (Chu et al., 2020). Nevertheless, the choice of method (e.g., regression equations) affects the accuracy of prediction, and the lack of detailed morphology means that the nose surfaces cannot be directly produced.

A landmark‐based GM method was developed to quantify the relationships between nasal hard and soft tissue morphologies. The examination of shape changes in the two blocks facilitates ambiguity avoidance in subjectively interpreting the relationship, and multiple regression algorithms can visualize and illustrate the morphological changes using TPS grids. Kustár et al. (2013) suggested a narrowing of nose width and deepening of the nose root correlated to a deeper nasal root and more projecting nasal margins and spin. Guyomarc'h et al. (2014) indicated there was a significant correlation (RV = 0.21) for the mid‐facial respiratory component of the French population and found that a thinner nose was related to a narrow face and nasal aperture. Since a limited number of landmarks may not capture the detailed biological structures, the present study used high‐density semilandmarks to capture the complete morphology of hard and soft tissues, although inaccurate identification of equivalence points might negatively affect the morphometric analysis (Cardini, 2020). A higher significant correlation (0.525 and 0.383 in the first three and all PLS) between hard and soft tissues and visualization of detailed morphological shape changes in the two blocks of PLS1 suggest the main features of nasal hard and soft tissues change together.

The landmark‐based GM method was also used to explore the covariations between oral hard and soft tissues. Malá et al. (2018) used landmarks located on the facial profile and suggested the upper lip showed a statistically significant covariation (RV = 0.14) with the maxilla. However, the detailed features of mouth morphology cannot be captured completely and illustrated. Therefore, the 3D landmark‐based method was used to analyze the correlation (RV = 0.25) and the results indicated lips tended to be related to loss of teeth and alveolar recession, and age affects the variation of mouth morphology (Guyomarc'h et al., 2014). Compared to these results, we used high‐density geometric morphometrics to examine the mouth relationship. The higher correlation (0.412 and 0.325 in the first three and all PLS) suggests bony structure appears to have an effect on the soft tissues in a nearly linear manner. For example, enlarged and protruding anterior teeth are correlated with a mouth having narrow and protruding upper and lower lips. We, therefore, acknowledge that high‐density semilandmarks seem to enhance the correlation between hard and soft tissues, and hence multiple regression can contribute to approximating the soft tissue shapes.

### 4.2 The computerized FA method

Anatomical modeling of craniofacial relationships is fundamental for the computerized FA method. Several existing FA methods (De Buhan & Nardoni, 2018; Deng et al., 2011; Shui et al., 2021; Turner et al., 2005) for representing craniofacial relationships in a holistic way probably cannot recreate the most accurate approximation, because the deformed facial features of the template head are treated as the approximation result and bony structures are not considered. It is acknowledged that different components exhibit different morphological changes in craniofacial relationships, for example, the correlations obtained are moderate for the mouth and nose, but relatively low in the region of eyes and ears (Guyomarc'h et al., 2014; Wilkinson, 2010). Therefore, we developed a coarse‐to‐fine strategy to approximate the soft tissues of each component separately and then recreated the whole approximated face. It is considered that this can facilitate improvements in the accuracy of the approximated face.

The virtual sculpture technique used FSTDs at landmarks to generate several facial points and predict muscle attachments (Wilkinson et al., 2006). Lee et al. (2015) concluded that average FSTDs at landmarks increase the accuracy of approximated faces. The present study used FSTDs at landmarks and semilandmarks to approximate the facial envelope with a close resemblance to the actual one, especially in regions exhibiting small FSTDs, for example, the forehead and scalp. This guarantees morphological consistency between the approximated face and dry skull and prevents overlap of the approximated face within bony structure. A small average approximation error indicates that dense FSTDs can contribute to representing overall craniofacial relationships and enhancing approximation accuracy, which is superior to the use of FSTDs at landmarks (Lee et al., 2015) and the regression‐based approach (Shui et al., 2020). However, the greatest variations can be observed around the cheeks, the chin, and temporal regions, consistent with the results of a previous study which showed that the FSTD variations were greatest around these regions (Dong et al., 2012). Hence, different distributions of FSTDs associated with age, sex, and BMI can be used to generate multiple facial appearances of the same skull.

As in previous studies (Guyomarc'h et al., 2014; Kustár et al., 2013; Ridel et al., 2020), the regression method was used to predict nose or mouth morphologies. Here we improve the multiple regression method and find that the choice of landmark configuration affects the approximated results. High‐density semilandmarks seem to better approximate nose and mouth shapes than landmarks alone, although the differences between them are relatively small. However, large ratios in approximated soft tissues can be observed, and indicate that nose and mouth shapes need to be very carefully produced. The potential reason is that soft tissues of facial features cannot be totally determined by bone morphology, for example, nasal protrusion seems less likely to be associated with the nasal bone (Kustár et al., 2013) and there were no statistically significant covariations between nasal bridge and tip and the lateral border of the nasal aperture (Malá et al., 2018). This may explain why the extent of artistic interpretation and imagination requires consideration to enhance detailed facial feature morphologies (Stephan, 2015; Wilkinson, 2010).

The previous study used landmark‐based GM to approximate ears and eyes (Guyomarc'h et al., 2014). The greater approximation biases in ears (7.0 mm) and eyes (2.9 mm) implied considerably weaker correlations between their hard and soft tissues. Hence, the covariations of landmarks and semilandmarks have not been explored in the present study. Instead, facial SSM is applied to predict the eye and ear morphologies with a prior knowledge as a constraint. The underpinning idea is to translate the question of predicting the eyes and ears into a geometric and statistical question, that is, the eyes and ears are estimated based on the other soft tissue morphology in the shape space. However, greater errors can be observed around the inner canthus of the eyes, and the helix and lobule of the ears, indicating that the approximation needs to be used with caution; The extent of artistic interpretation to show detailed facial feature morphologies requires careful consideration.

A quantitative assessment enables us to validate the accuracy of the proposed FA method and to continue development and improvement, focusing on regions with the greatest discrepancies. In recent years, the most frequently used method for quantitative assessment is to compare the geometric difference between approximated and actual faces (Stephan & Henneberg, 2006). Several protocols, for example, shell‐to‐shell or surface‐to‐surface deviation, have been employed to compare approximated and actual faces (Decker et al., 2013; Lee et al., 2012; Miranda et al., 2018; Short et al., 2014). A straightforward method for superimposing these two faces is to perform registration based on at least three landmarks, thereby visually observing the profile variations and computing the geometric difference. Because the registration results greatly affect the evaluation of the level of approximation error and resemblance, they need to be carefully examined before performing assessment. In the present study, the landmarks and semilandmarks covering the entire head are used to register the approximated and actual faces, and then the gross and regional differences are recognized. To avoid dependencies on the dataset, we suggest the same test cases, dataset, and evaluation method should be used for validating the level of effectiveness of different FA methods.

### 4.3 Limitations and future work

Landmarks describe the shape and size of biological specimens and they are always in terms of anatomical, developmental, biomechanical, or evolutionary knowledge (O'Higgins, 2000). However, only a few landmarks can be readily identified over smooth surfaces such as the scalp. In morphometrics, semilandmarks determined by algorithms have increasingly been applied to capture detailed morphology. The choice of landmark and semilandmark configuration affects the morphometric analysis and visualization (Shui et al., 2023). This study is limited in its scope, having examined landmarks alone and landmarks and high‐density semilandmarks together to explore nose (or mouth) covariations between hard and soft tissues and to predict soft tissue shapes. Future work should consider an examination of different configurations with regard to semilandmark location and density on the covariation analysis and soft tissue approximation. Additionally, numerous studies have recorded FSTDs at landmarks associated with sex, age, and BMI (De Greef et al., 2009; Dong et al., 2012), and explored the covariations between hard and soft tissues dependent on personal information (Ridel et al., 2020). In the present study, age and sex effects are not investigated due to the relative homogeneity of the test population. Further study is needed to assess the effect of using varied factors (e.g., sex, age, and BMI) as independent variables on the craniofacial relationships of facial features, thereby improving the accuracy of the approximated soft tissue shapes.

## 5 CONCLUSION

This study developed a computerized FA method for exploring craniofacial relationships and developing a coarse‐to‐fine strategy for generating facial appearances. Average FSTDs at landmarks and semilandmarks can be used to quantify the overall craniofacial relationship and contribute to enhancing the accuracy of the approximated face. Nasal and oral hard tissues have an effect on their soft tissue shapes separately, and hence the multiple regression can be used to approximate the nose and mouth soft tissue shapes. However, relatively weaker covariations and greater approximation errors suggested the approximated nose and mouth should be considered with caution. This method should be useful for a broad range of applications in forensic science, anthropology, and archaeology.

## AUTHOR CONTRIBUTIONS

Author contributions: Conceptualization, Shui, Wu, and Zhou; methodology, Shui; software, Shui; validation, Shui; formal analysis, Shui; investigation, Shui, Wu, and Zhou; resources, Shui, Wu, and Zhou; data curation, Shui, Wu, and Zhou; writing—original draft preparation, Shui; writing—review and editing, Shui, Wu and Zhou; visualization, Shui; Funding, Wu.

## CONFLICT OF INTEREST STATEMENT

The authors declare that they have no competing interests.

## ETHICS STATEMENT

This study was approved by the Ethics Review Committee of the Department of Archaeology, University of York (Date: 20th April 2021).

## CONSENT

We declare that written informed consent was obtained from all participants included in the study.

## Data Availability

The datasets used and analyzed during the current study are available from the corresponding author on reasonable request.
